# Supplementary material for: Identifying the Trends of Urinary microRNAs within Extracellular Vesicles for Esophageal Cancer
Source: Cancers (Basel). 2024 Apr 27;16(9):1698. doi: 10.3390/cancers16091698 (PMC11083496; doi:10.3390/cancers16091698)

Supplemental Figure

Supplemental Figure S1. Trends of microRNAs during multidisciplinary treatment: Case 1.

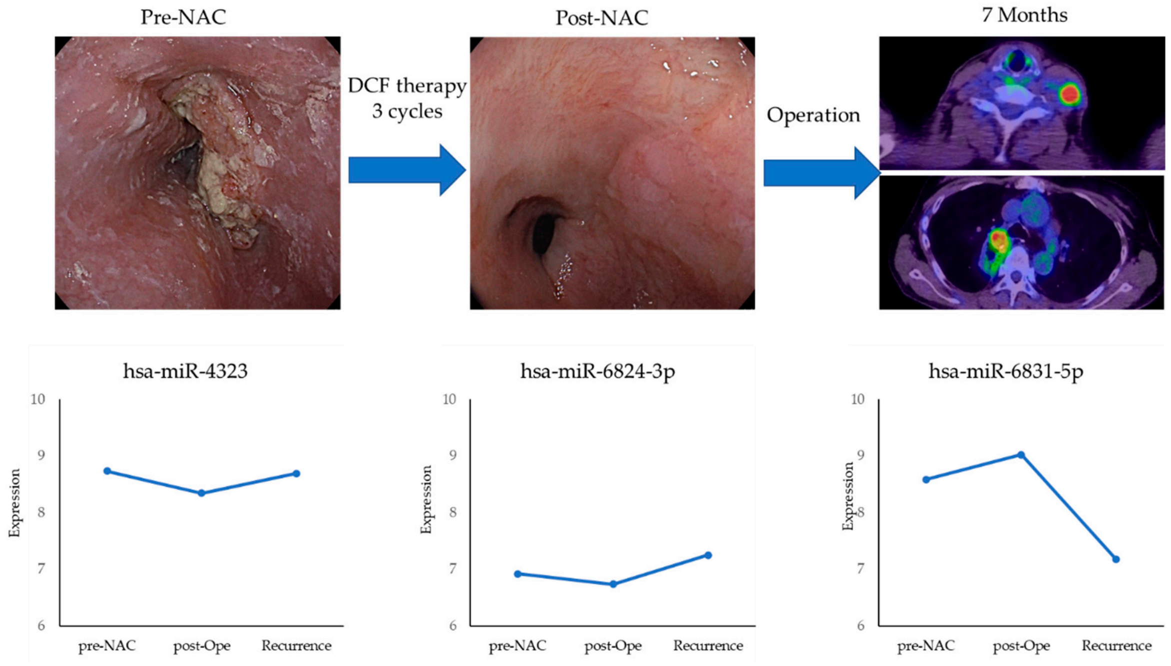

Supplemental Figure S2. Trends of microRNAs during multidisciplinary treatment: Case 2

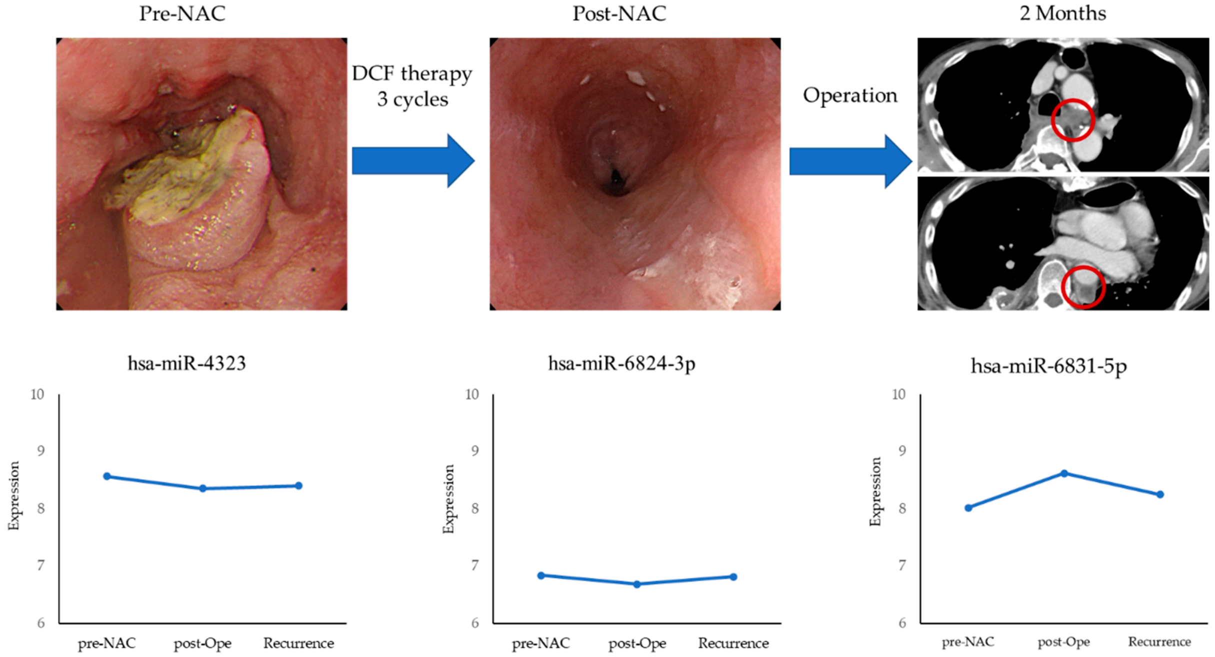

Supplemental Figure S3. Longitudinal Changes in microRNAs in recurrence case 1

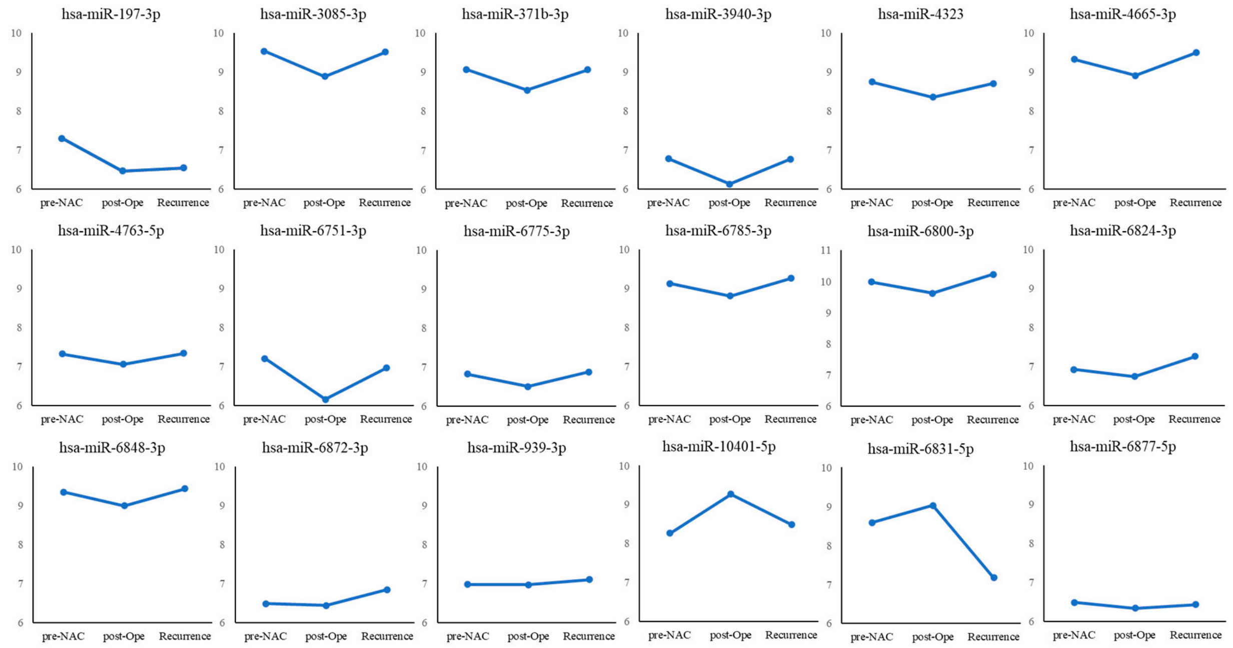

Supplemental Figure S4. Longitudinal Changes in microRNAs in recurrence case 2

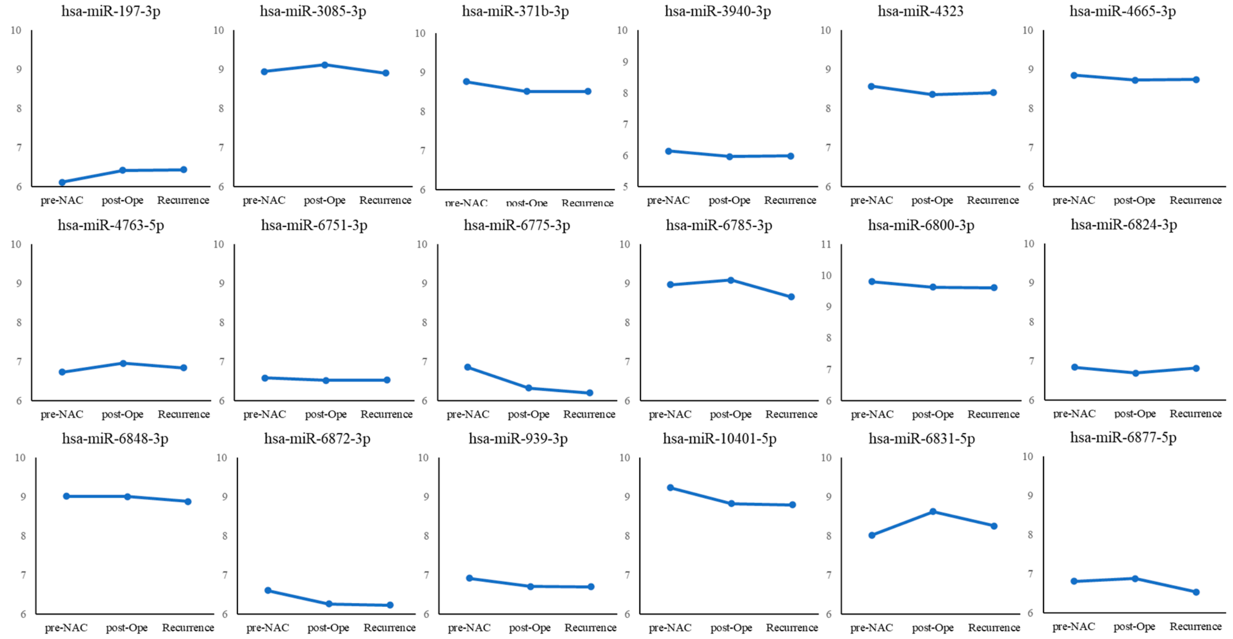

Supplement: Supplementary file 1 [file cancers-16-01698-s001.zip › cancers-2902475-supplementary.pdf]
